# A complex intervention on vaccination uptake among older adults (≥ 60 years) in Germany – a study protocol with a mixed methods design

**DOI:** 10.1186/s12875-023-02101-w

**Published:** 2023-07-15

**Authors:** Sarah A. K. Uthoff, Anna Zinkevich, Dominika Franiel, Maike Below, Helene Splieth, Julia Iwen, Marc Biedermann, Dorothee Heinemeier, Lena Ansmann

**Affiliations:** 1 Department of Health Services Research, Faculty of Medicine and Health Sciences, Carl von Ossietzky University of Oldenburg, Ammerlaender Heerstrasse 140, 29123 Oldenburg, Germany; 2 Institute of Medical Sociology, Health Services Research, and Rehabilitation Science (IMVR), Chair of Medical Sociology, Faculty of Medicine and University Hospital Cologne, University of Cologne, Eupener Str. 129, 50933 Cologne, Germany; 3 Central Research Institute of Ambulatory Health Care in Germany, Salzufer 8, 10587 Berlin, Germany; 4 Association of Substitute Health Funds (Vdek) e.V, Askanischer Platz 1, 10963 Berlin, Germany; 5 The National Association of Statutory Health Insurance Physicians, Herbert-Lewin-Platz 2, 10623 Berlin, Germany; 6 Communication Lab Erfurt, Bahnhofstraße. 16/Büßleber Gasse, 99084 Erfurt, Germany

**Keywords:** Preventive health, Elderly care, Vaccination uptake, General practice, Primary care, Influenza vaccination

## Abstract

**Background:**

The current uptake of many vaccinations recommended for persons aged 60 and older is unsatisfactory in Germany. Lack of confidence in the safety and efficacy of vaccinations, lack of knowledge and insecurities about possible side effects, and numerous pragmatic barriers are just some of the reasons to be mentioned. General practitioners (GPs) play a central role in the vaccination process. Therefore, effective interventions in this context are needed to address the various barriers and improve the vaccination uptake rates.

**Methods:**

A complex intervention will be implemented and evaluated in 1057 GPs’ practices in two German federal states. The components include trainings for GPs and medical assistants on communication psychology, medical aspects, and organisational vaccination processes. The primary outcome influenza vaccination rate and the secondary outcomes vaccination uptake rate of other vaccinations as well as vaccine literacy of patients will be examined. The intervention will be evaluated in a mixed methods study with a controlled design. Survey data will be analysed descriptively and by using mean comparisons as well as multivariable multilevel analyses. The qualitative data will be analysed with qualitative content analysis. The secondary data will be analysed by using descriptive statistics, a pre-post comparison by performing mean comparisons, cluster analysis, and subgroup analyses.

**Discussion:**

In this study, a complex intervention to improve vaccination rates in GP practices for the vaccinations recommended for people aged 60 years and older will be implemented and evaluated. Additionally, improvements in patients’ vaccine-related health literacy and knowledge, and patients’ intention to get vaccinated are expected. The mixed methods design can deliver results that can be used to improve preventive health care for elderly people and to gain more knowledge on vaccination uptake and the intervention’s effectiveness.

**Trial registration:**

Trial registration number: DRKS00027252 (retrospectively registered).

**Supplementary Information:**

The online version contains supplementary material available at 10.1186/s12875-023-02101-w.

## Background

With older age the prevalence of chronic conditions such as diabetes mellitus or cardiovascular problems is increasing. At the same time, the age-associated weakening of the immune system contributes to an increased incidence and severity of infectious diseases. Therefore, severe courses or life-threatening complications from infections occur more frequently with older age [1–4]. Infections such as seasonal influenza can therefore last longer or run a more severe course, with serious health risks such as pneumonia or myocarditis. In addition, the risk of secondary diseases (e.g., sepsis as a result of influenza or pneumococcal infection) is higher in patients aged 60 and older. There is also a significant increase in mortality as well as hospitalization among elderly people infected with influenza [5]. The COVID-19 pandemic also shows the increased vulnerability of people in older age to new pathogens [6, 7]. For these reasons, the uptake of existing vaccinations for patients aged 60 and older is of major relevance for preventing the aforementioned severe consequences.

In Germany, the current uptake of vaccinations that are recommended for this age group according to the Standing Committee on Vaccination (STIKO; seasonal influenza, pneumococci, herpes zoster and revaccinations against tetanus, diphtheria and pertussis) is unsatisfactory [8]. In particular, the annual influenza vaccination rate is low in the age group 60 and older (influenza season 2020/2021: 47.3%) and far from the rate of 75% recommended by the Council of the European Union [8, 9]. The rates for pneumococcal vaccination in Germany are low and vary widely between 14.8% and 40.4% depending on the region in the age group between 60 and 73 years [8]. The vaccination rate against herpes zoster in the German population over 60 years is generally extremely low, in the single digits [8].

The reasons for the rather low vaccination rates in Germany in European and international comparison are multifactorial [10–12]. Numerous studies show that a number of psychological, contextual, sociodemographic, and pragmatic barriers to influenza vaccination uptake exist. Lack of trust, inconvenience, calculation, and complacency are reported to be barriers to influenza vaccination in risk groups to varying degrees [13]. In addition to a lack of trust in the safety and effectiveness of vaccinations and incomplete knowledge about possible side effects [14, 15], a misconception of vulnerability to certain diseases and their effects on health in the elderly is also a significant reason for the low uptake. Furthermore, a low sense of responsibility towards the community as well as a lack of understanding with regard to the necessary herd immunity also have an influence on individual vaccination motivation and uptake. In addition, numerous pragmatic barriers exist on the part of patients and general practitioners (GPs). These are, for example, lack of time, access to GP practices or forgetfulness [13, 16]. In Germany, vaccinations are usually provided by GPs [17]. Studies show that physicians’ recommendations are an important influencing factor that patients are aware of vaccinations [13]. The mentioned diverse reasons for the individual decision to get vaccinated are summarised within the following domains of the 5 C model: confidence, complacency, constraints, calculation, collective responsibility [16, 18]. These domains apply not only to the elderly, but to the general population. Effective measures are needed to address these diverse barriers. For example, effective communication strategies and techniques used by GPs during the encounter can help improve education and counselling and thereby increase patients’ trust in vaccinations and relevant knowledge. Studies show that by using motivational interviewing, emerging concerns and fears can be addressed empathically and precisely [19]. When dealing with vaccination myths and misinformation, it is important to use targeted communication and to address the techniques used, for example, in vaccine-sceptic argumentation [20, 21]. For GPs, it is also important to know the reasons for non-vaccination and to be aware of vaccination fatigue. Structural barriers can be effectively addressed by implementing vaccination recall systems – a procedure that has already achieved positive effects in numerous studies [22, 23]. In this context, medical assistants in GP practices have an essential function. They are the first point of contact for patients and are responsible for administrative practice procedures. It can therefore be important to integrate medical assistants into the vaccination process and, after appropriate training, to give them responsibility for vaccination management [24, 25].

### Description of the complex intervention

Against the aforementioned background, a bundle of measures to increase vaccination uptake will be implemented in GP practices. The aim of the intervention is to raise further awareness among GPs and medical assistants about the importance of vaccinations for patients aged 60 and older, to provide them with training in vaccination expertise and communication psychology, to introduce suitable tools for vaccination recall into their practice routines, and to update them on where to find target group appropriate information for patients.

As the first component of the intervention, GPs and their medical assistants complete an accredited online training. The main goal of the online training is to increase medical knowledge about vaccinations and vaccination counselling skills. This is intended to promote vaccine-related communication between GPs and patients, improve patient vaccine literacy, and thereby increase vaccination uptake. Furthermore, participating GP practices will receive a first material package containing appointment reminder cards, a poster on vaccination, checklists on the vaccination procedure in outpatient care and nursing homes, an infosheet with the most important contents from the online training, an immunisation schedule with an overview of recommended vaccinations, and templates of consent forms for vaccination reminders for the following influenza vaccination season.

**Fig. 1:**
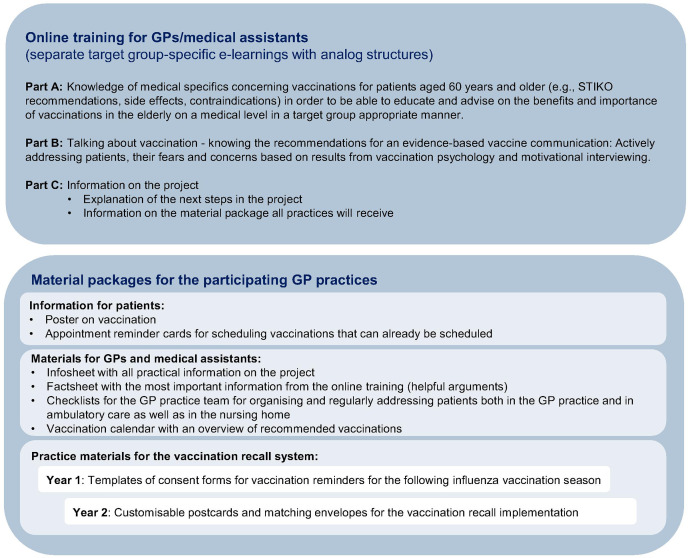
Components of the complex intervention

In the second year of the intervention, the GP practices will again receive a second material package before the start of the influenza vaccination period. This contains, among other things, the materials needed for the upcoming vaccination recall (customisable postcards and matching envelopes). The intervention is planned to be implemented in the GP practices from 01.07.2022 to 31.03.2024. Figure 1 shows the components of the complex intervention.

### Aims of the study

**Fig. 2:**
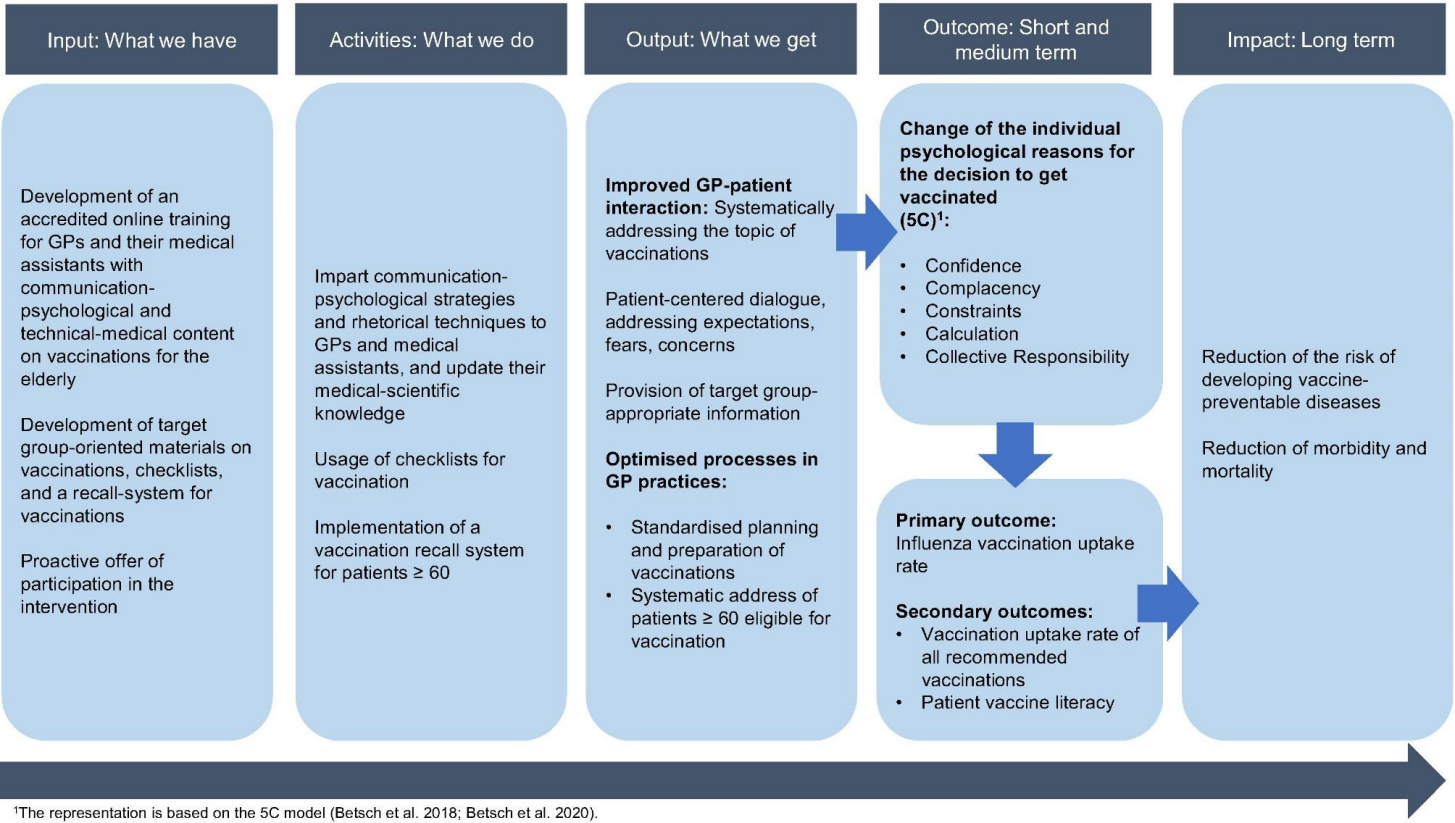
Logic model of the complex intervention based on Moore et al. (2015)

The main aim of the study is to develop, implement and evaluate a complex intervention to significantly improve vaccination rates for the vaccinations recommended by the STIKO for persons aged 60 and older (seasonal influenza, pneumococci, herpes zoster, COVID-19, and revaccinations against tetanus, diphtheria, and pertussis). The following logic model (Fig. 2) demonstrates how the intervention is intended to work and how it is assumed to affect the intended results according to the MRC framework [26].

## Methods and analysis

### Study design

Not only the effect on outcomes (summative evaluation), but also the process of implementing the intervention (formative evaluation) must be examined in order to identify mechanisms of action and contextual factors, as this is a complex intervention [27].

### Summative evaluation

Within the summative evaluation, secondary as well as primary data will be used (see Table 1).

#### Summative evaluation: secondary data

The secondary data analysis is based on data on vaccination rates before and after the intervention, which will be compared between intervention and control group and will address the following research questions:

1. Does the intervention lead to an increase in influenza vaccination uptake rates in the GP practices among persons insured by one of the six substitute health insurance funds aged 60 and older (primary outcome) group?
2. Does the intervention lead to an increase in the vaccination uptake rate for the other vaccinations recommended by the STIKO for persons aged 60 and older (seasonal influenza, pneumococci, herpes zoster, and revaccinations against tetanus, diphtheria, and pertussis) (secondary outcome)?
3. Since previous analyses of vaccination activity have shown significant regional differences in vaccination uptake rate [28], do the effects in (1) and (2) vary between the participating regions of Westphalia-Lippe, North Rhine and Schleswig-Holstein?

The focus of the summative evaluation will be on a pre-post comparison between the intervention and control group, considering differences between the two groups that already existed before the intervention. For the post analysis, GP claims data from 7 quarters after the start of the intervention are available for analysis (07/01/2022–03/31/2024). Due to the still unknown impact of the COVID-19 pandemic on the next influenza vaccination season, a period of at least 24 months prior to the start of the intervention will be considered for pre-analysis – meaning the vaccination and immunisation uptake rates achieved prior to the intervention. Because influenza vaccination should occur annually, comprehensive information on the baseline population and the number of vaccinated persons is available. The determination of vaccination uptake rates, on the other hand, is more challenging because the size of the population is unclear due to the long periods of time between booster vaccinations. Persons aged 60 and older are recommended to receive a booster vaccination against tetanus, diphtheria, and pertussis only every 10 years. A booster vaccination against pneumococci after 6 years (or later) is beneficial but not generally recommended by the STIKO. Furthermore, there is no recommendation for a booster vaccination against herpes zoster after the basic immunisation has been completed. The available data does not allow to identify if patients aged 60 and older got a booster vaccination against tetanus, diphtheria, and pertussis in the past 10 years and if patients aged older than 60 have already been vaccinated against pneumococci or herpes zoster. Therefore, we will analyse the increase in the vaccination uptake rate among participating GPs compared to non-participating GPs. Provided that the necessary number of cases is achieved, subgroup analyses for individual vaccinations (e.g., pneumococci) are planned.

#### Summative evaluation: primary data

The primary data for the summative evaluation will be collected by two standardised cross-sectional patient surveys in the intervention group pre and post implementation. Patients who visited the GP practice before implementation of the intervention act as a pre-implementation baseline. After implementation, a second patient survey will be conducted in the same GP practices (post implementation). The summative evaluation using primary data addresses the following research question:

1. Do patients in GP practices post-implementation report better vaccine literacy than patients pre-implementation?

Vaccine literacy is defined as a secondary outcome, as an association with vaccination uptake is known from the literature [29, 30].

### Formative evaluation

The formative evaluation is conducted using primary data and will examine various elements of the intervention and its implementation [26, 27].

#### Formative evaluation: primary data

The formative evaluation aims to answer the following questions:

1. How do the GPs and medical assistants rate the feasibility and usefulness of the intervention components? Which components need improvements?
2. Have vaccination procedures improved in GP practices from the perspective of GPs and medical assistants, and which intervention components are assessed as effective?
3. How do patients perceive the information and motivation for vaccination in the GP practices, especially in the GP-patient communication?

The formative evaluation is designed as an accompanying observational study in which the perspectives of the patients, the GPs, and medical assistants on the intervention and its implementation will be explored using qualitative interviews (with patients, GPs, and medical assistants) and quantitative surveys (patients, GPs, and medical assistants).

### Sample

Estimating the effect on vaccination uptake rates to be achieved by the complex intervention is not trivial. From meta-analyses, it is known that effects of around 5% can be achieved by campaigns or interventions that distribute information in a study group, e.g., in the form of brochures, billboard advertisements, flyers, postcards, newsletters, but also by interpersonal communication between GP and patient as well as community education (seminars at schools, workplace) [31]. However, it must be considered that the study results known so far from the literature do not explicitly focus on vaccination. It is not yet known how the COVID-19 pandemic will affect vaccination uptake of established STIKO vaccines in the short and medium term. Due to the above-mentioned, hardly calculable development and the unpredictable development in GP practices, the achievable effect of the project must be estimated conservatively. This is even more important since the data used to estimate the number of cases reflect the situation before the pandemic.

Thus, it is assumed in the following that the influenza vaccination uptake rate as primary outcome increases by 3% as a result of the intervention. This 3%-increase should be seen as an initial change. To detect this effect in the secondary data analysis with a power of 90% at a significance level of alpha = 5% (two-sided), 17,508 patients would be needed without considering the cluster effect. Based on available anonymised data from previous studies [32, 33], the intra-cluster correlation (ICC) is expected to be 0.08. With a mean cluster size of 48 patients per practice vaccinated against influenza on average, this results in a design effect of 4.76 and a corresponding required case number of 83,338 patients. Accordingly, about 1736 GPs would be needed (868 GPs in the intervention and control groups each). Likewise, an increase of 3% is also targeted for increase in vaccination uptake rate of further recommended vaccinations as the secondary outcomes. Since the assumed cluster size is reduced to 28, resulting in a design effect of 3.16 with an ICC of 0.08, a case number of 55,325 patients is required for these analyses. For this, 1976 GPs are needed according to the cluster size – 988 GPs in each of the intervention and control groups. Therefore, due to the smaller cluster size for the secondary outcome, a total of 988 GPs is required for the intervention group for the analysis. To ensure that the required number of cases will be achieved, an additional 7% (69 GPs) was added to the calculated number of cases. This resulted in a required total number of 1057 GPs per group.

### Recruitment

The recruitment of GPs for study participation takes place three months prior to the start of the intervention phase by the three participating regional Associations of Statutory Health Insurance Physicians (ASHIP) Schleswig-Holstein, North Rhine and Westphalia-Lippe, considering the available contingent of participants in their region. In Germany, the ASHIP are bodies under public law, which represent all physicians and psychotherapists treating compulsory insured patients. First, all potentially eligible GPs in the three regions, meaning GPs participating in primary care according to § 73 (1a) SGB V (excluding paediatricians), who have vaccinated patients aged 60 and older insured by the substitute health insurance fund in the past, will be identified. Substitute health insurance funds are a type of health insurance fund with the largest percentage of insured persons on the German market. There are no other major differences between this and other types of health insurance funds. Subsequently, these GPs will be stratified by the three ASHIPs, and randomised into two groups (intervention/control). The potential participating GPs in the intervention group will be contacted by the responsible ASHIP and receive the offer to participate in the intervention group. It is pointed out that participation in the intervention is also associated with participation in the accompanying evaluation study. Subsequently, all enrolled GPs, as well as at least one medical assistant from the practice, complete the online training that is completed after an online knowledge test and submit the certificate of successful participation to the associated ASHIP.

#### Secondary data

After submitting the certificate and vaccinating at least 20 patients aged 60 and older insured by a substitute health insurance fund in the first quarter of evaluation, the GP will receive an initial starter lump sum payment. Through this lump sum payment, the GP is marked as a participant in the billing data of the ASHIP and is available in a pseudonymised form for the summative evaluation over all quarters considered. Since secondary data (extract of pseudonymised contractual GP billing data according to § 295 SGB V) is used, the relevant data is available for both the intervention and the control group.

#### Primary data

##### Pre-post patient survey

The patient survey will be conducted in n = 50 randomly selected intervention GP practices that will be drawn from the population of n = 1057 GPs of the intervention group, stratified by ASHIP. The selected practices will receive the study materials for the two patient surveys pre and post implementation. The recruitment of patients will be done by the practice staff. Patients will be asked to complete an anonymous, paper-based questionnaire after their consultation with the GP. The questionnaires will be handed out in sealed envelopes and can be completed in the GP practice or at home. For the pre-implementation survey, patients who visited the GP practice in the 4–8 weeks before the intervention starts will be recruited. For the post-implementation survey, patients who visit the GP practice in the end of the intervention will be recruited. Each of the GP practices participating in the patient survey is asked to recruit a minimum of n = 25 patients for each survey round (2x n = 1250 patients). To increase the response, the practices receive a material incentive in the form of a gift box after n = 10 questionnaires and for all further questionnaires a financial incentive of 5 € per questionnaire.

##### Online survey of GPs and medical assistants

The recruitment for the online survey of GPs and medical assistants in all intervention practices (n = 1057 GPs and n = 1057 medical assistants) takes place via a postal letter to the GPs and the medical assistants. The cover letter contains a QR code to the online survey. To increase the response, a financial incentive (25 € per completed online survey) and other methods (e.g., 2 waves of reminders, personalised cover letters, university logo) are planned [34–36]. Based on previous experience from a similar project, a response of 66% (n = 698 GPs and n = 698 medical assistants) can be assumed.

##### Qualitative interviews with patients, GPs, and medical assistants

For the individual interviews, GPs and medical assistants in the participating GP practices will be recruited by telephone by the research team using purposeful sampling [37] (by gender, age, region, and individual vs. joint practice). GPs and medical assistants will be recruited preferably from the 50 intervention practices participating in the patient survey to enable integrated analyses of quantitative and qualitative data from the same practices. The recruited GPs and medical assistants will receive information on the study and consent forms in advance. The interviews can take place in the GP practice, at a location of their choice, or by telephone and will be audio-recorded. For the interview, which will last up to 60 min, a financial incentive is planned (50 € per interview).

For the patient interviews, participants will be recruited via the participating intervention practices (n = 50) using purposeful sampling (by gender, age, and region). The intervention GP practices will approach potential interview participants and will invite them to participate in the interview study. The research team will contact the patients and will arrange an interview appointment. The interviews will be conducted by the research team in the patients’ homes, at a location of their choice, or by telephone, and will be audio-recorded.

The estimated number of interviews in the three groups aligns with the recommendation of 20 to 30 interviews per interviewed group suggested by Flick et al. [38]. Accordingly, 30 interviews with patients, 20 interviews with GPs, and 20 interviews with medical assistants are planned. The higher number of interviews with patients is due to the fact that a higher heterogeneity, e.g., in terms of socio-demographic characteristics is expected. Flick [39] advocates the number of cases mentioned in order to obtain manageable and useful data.

### Measures

#### Secondary data collection

The influenza vaccination rate as primary outcome is measured annually for patients insured by a substitute health insurance fund aged 60 and older. The rate will be computed as the fraction of the amount of vaccinated patients insured by a substitute health insurance fund aged 60 and older and all patients insured by a substitute health insurance fund aged 60 and older of the practice under consideration.

The determination of the secondary outcome, vaccination uptake rates for vaccinations recommended by the STIKO for patients aged 60 and older (except for influenza), is more challenging. This is due to the unclear size of the basic population, which, in turn, is a result of the long periods of time between recommended booster vaccinations (the boosters for tetanus, diphtheria, and pertussis are needed/recommended only every 10 years).

If a sufficiently high number of cases is reached, subgroup analyses for different vaccinations are planned (e.g., for pneumococci and herpes zoster).

#### Primary data collection

In the pre-post patient survey, the secondary outcome vaccine literacy and further constructs such as general health literacy, psychological antecedents of vaccination behaviour, intention to get vaccinated, knowledge about vaccination, as well as numerous confounders and sociodemographic information will be assessed. The outcome vaccine literacy is assessed using the validated subscale of the Health Literacy Population Survey Project 2019–2021 (HLS19) instrument (HLS19-VAC-DE) [40]. General health literacy is measured with the generic short version of the instrument for measuring health literacy in the general population (HLS19-Q12; [40]. Psychological antecedents of vaccination behaviour are surveyed with the short version of the associated validated 5 C scale [18, 41]. The intention to get vaccinated is surveyed in a vaccine-specific manner using the item from the study by Betsch et al. [42].

Knowledge about influenza and pneumococci vaccination is assessed using an instrument based on the validated scale developed by Zingg & Siegrist [43]. (Un)met information needs regarding vaccination are assessed with items based on an instrument developed by Neumann et al. [44]. In addition to the listed central constructs, possible moderators such as the GPs’ empathy (Consultation and Relational Empathy scale) [45, 46] and trust in the GPs and medical assistants [47] are also included in the questionnaire. Furthermore, patient experiences regarding communication with the practice staff are surveyed. These include aspects such as carrying out a vaccination check, reminding, answering questions regarding vaccinations, and distributing written information. Sociodemographic variables (e.g., age, gender, employment) will be collected at both time points (pre and post implementation), as this is an anonymous survey with independent patient populations.

The focus of the online survey of GPs and medical assistants is on their experiences with and evaluation of the intervention. These aspects will be assessed with both self-developed instruments and validated instruments. For assessing the self-reported communication competence, the validated scale Medical Communication Competence Scale [48] is used. Furthermore, the online survey will include questions on structural characteristics of the GP practices such as size and type of the practice as well as sociodemographic characteristics of the GPs and the medical assistants (e.g., age, gender).

For the formative evaluation, guideline-based, semi-structured interviews with GPs, medical assistants, and patients will be conducted. In the interviews, patients will be asked about their vaccine literacy, their attitudes toward vaccination, their knowledge about vaccination, and their experiences regarding vaccine-related communication with the GP and the medical assistant (see Fig. 2). In the interviews, the GPs and medical assistants will mainly be asked about their experiences with the intervention and their vaccine-related communication with the patients.

**Table 1:** Data collection methods for the summative and formative evaluation

Data collection methods and timepoints	Indicators to be measured	Measurement instruments	Data analysis
***Summative evaluation (secondary and primary data)***			
**Secondary data**: Extract of pseudonymised contract GP billing data according to § 295 SGB V, n = 2114 GPs (n = 1057 GPs each in the intervention and control group) and at full utilisation ≥ 147,980 patients	**Primary outcome**: Influenza vaccination rate **Secondary outcome**: Vaccination uptake rate of the vaccinations recommended by the STIKO for patients aged 60 and older (except for influenza)	-	Descriptive statistics, pre-post comparison (intervention/control group), cluster analysis, subgroup analyses
**Primary data**: Cross-sectional patient survey in intervention practices (n = 50), pre- and post-implementation (2 x n = 1250 patients), paper & pencil survey	**Secondary outcome**: Vaccine literacy	**Further secondary outcome**: HLS19-VAC-DE subscale of the HLS19 instrument (40)	Descriptive statistics, multivariable regression models, multilevel analysis
***Formative evaluation (primary data only)***			
Cross-sectional patient survey in intervention practices (n = 50), pre- and post-implementation (2 x n = 1250 patients), paper & pencil survey	Attitudes towards vaccination; intention to get vaccinated; knowledge about vaccination; (un)met information needs; vaccination-related interaction between GP practice staff and patients	5 C instrument (18, 41); vaccination intention item (42); instrument based on the validated scale developed by Zingg & Siegrist (43) to measure knowledge about vaccination; instrument based on CaPts to measure (un)met information needs (44); self-developed items on vaccination-related interaction between GP practice staff and patients	Descriptive statistics, multivariable regression models, multilevel analysis
Cross-sectional online survey with GPs and medical assistants in intervention practices (n = 2 × 1057)	Experiences with and evaluation of the intervention elements, also in nursing homes and outpatient care, evaluation of changes in the GP-patient interaction; self-reported communication competence of GPs; structural information related to GP practices	Self-developed, best-fit instruments on experience with and assessment of the intervention; Medical Communication Competence Scale (MCCS; (48); self-developed items on structural information related to GP practices	Descriptive statistics, multilevel analysis after linkage with patient survey data, subgroup analyses
Qualitative interviews with patients in intervention GP practices, n = 30	Experiences with the intervention, subjective effectiveness	Semi-structured guideline	Structuring qualitative content analysis according to Kuckartz (49)
Qualitative interviews with GPs/medical assistants in intervention GP practices, n = 40	Experiences with the intervention, subjective effectiveness	Semi-structured guideline	Structuring qualitative content analysis according to Kuckartz (49)

### Data analysis

#### Secondary data

As the current COVID-19 pandemic had a considerable effect on influenza vaccination rates and at least on pneumococci vaccination rates, too, it is important to differentiate between a baseline before the pandemic and a baseline during the pandemic. The alterations in vaccination rates due to the pandemic will be determined by using the Interrupted Time Series Models (ITS).

After the determination of the baselines, pre-post comparison analyses will be conducted for both outcomes differentiated by intervention and control group. The pre-post comparisons will be conducted by performing comparisons of means based on two-sided tests with significance levels of 5%. Under consideration of clustering, various influencing factors are taken into account.

Furthermore, subgroup analyses such as for patients in nursing homes or for single vaccinations such as the pneumococci vaccination are planned. Finally, if a sufficiently high number of cases in all regions is reached, a comparing analysis of the vaccination uptake rates between the three regions will be conducted.

#### Primary data

The paper questionnaires will be scanned using the Electric Paper TeleForm software, in which they are subjected to detailed plausibility tests. The primary data will be analysed using descriptive statistics, tests for mean differences in independent samples and multivariable multilevel analyses to combine GP practice-level data from the online survey with individual patient survey data. All individual interviews will be audio-recorded and then transcribed and pseudonymised. The transcripts will be analysed with the structuring qualitative content analysis according to Kuckartz [49] using the software MAXQDA Analytics Pro. First, main categories will be developed a priori on the basis of the interview guideline, and then inductive subcategories will be developed during the coding process. The coding process will be carried out independently by two researchers with a subsequent consensus-finding procedure.

#### Patient and public involvement

The project does not include any genuine patient and public involvement components. However, GPs are represented within the project team by the participating ASHIP. A representative of the German Association of Medical Assistants is part of the consortium as well and contributed to intervention development.

## Discussion

In this study, a complex intervention to improve vaccination rates in GP practices for the vaccinations recommended for people aged 60 years and older will be implemented and evaluated. Targeted intervention activities in the GP practices are intended to improve patients’ vaccine-related health literacy, knowledge about the respective diseases and vaccinations, and patients’ intention to get vaccinated.

The survey recruitment of patients by the GP practice team may lead to a social desirability bias. Since independent patient cohorts are surveyed pre and post intervention, no within-person comparisons can be performed. Furthermore, the dynamic developments of the COVID-19 pandemic could affect both outcomes and the feasibility of the planned study.

To the best of our knowledge, there are no comparable complex intervention studies examining vaccination uptake and vaccine literacy regarding all recommended vaccinations for persons aged 60 years and older in Germany. Therefore, the design of this intervention study can deliver results that can be used to improve preventive health care for elderly people. The mixed methods design enables a more comprehensive understanding of the complex context in which this study takes place and thus contributes to more profound knowledge on the intervention’s effectiveness.

The results will be published as a report in the final phase of the study. Depending on the results, a decision will be made by the Innovation Fund of the Federal Joint Committee, the highest decision-making body of the joint self-government of physicians, dentists, hospitals, and health insurance funds in Germany on whether the intervention will be recommended to be included into standard care in Germany. The results will be accessible to the scientific community in the form of scientific publications and conference contributions. Aggregated results of the patient surveys per GP practice can be provided to participating GPs on request.

## Electronic supplementary material

Below is the link to the electronic supplementary material.

Supplementary Material 1

## Data Availability

The datasets used and/or analysed during the current study available from the corresponding author on reasonable request.

## List of abbreviations

GP: General practitioner
STIKO: Standing Committee on Vaccination
ICC: Intra-cluster correlation
ASHIP: Associations of Statutory Health Insurance Physicians
SGB: V Fifth Social Security Code
HLS19: Health Literacy Population Survey Project 2019–2021
CARE: Consultation and Relational Empathy scale
MCCS: Medical Communication Competence Scale
CaPts: Information needs of cancer patients scale
ITS: Interrupted Time Series Models
EU: GDPR General Data Protection Regulation
vdek: Verband der Ersatzkassen e. V [Association of Substitute Health Funds]
